# A facile heterogeneous system for persulfate activation by CuFe_2_O_4_ under LED light irradiation†

**DOI:** 10.1039/c9ra05574f

**Published:** 2019-10-10

**Authors:** Xin Zhong, Xiao-Yu Ye, Di Wu, Kai-Xin Zhang, Wei Huang

**Affiliations:** Department of Environment Science and Engineering, Beijing Normal University Zhuhai 519000 China zhongxin@whu.edu.cn

## Abstract

In this study, the removal performance for rhodamine B (RB) by persulfate (PS) activated by the CuFe_2_O_4_ catalyst in a heterogeneous catalytic system under LED light irradiation was investigated. The effect of vital experimental factors, including initial solution pH, CuFe_2_O_4_ dosage, PS concentration, co-existing anion and initial RB concentration on the removal of RB was systematically studied. The removal of RB was in accordance with the pseudo first-order reaction kinetics. Over 96% of 20 mg L^−1^ RB was removed in 60 min using 0.5 g L^−1^ CuFe_2_O_4_ catalyst and 0.2 mM PS at neutral pH. In addition, free radical quenching experiments and electron spin resonance (EPR) experiments were performed, which demonstrated the dominant role of sulfate radical, photogenerated holes and superoxide radical in the CuFe_2_O_4_/PS/LED system. The morphology and physicochemical properties of the catalyst were characterized by XRD, SEM-EDS, TEM, N_2_ adsorption–desorption isotherm, UV-vis DRS, and XPS measurements. Moreover, 18.23% and 38.79% total organic carbon (TOC) removal efficiency was reached in 30 min and 60 min, respectively. The catalyst revealed good performance during the reusability experiments with limited iron and copper leaching. Eventually, the major intermediates in the reaction were detected by GC/MS, and the possible photocatalytic pathway for the degradation of RB in the CuFe_2_O_4_/PS/LED system was proposed. The results suggest that the CuFe_2_O_4_/PS/LED system has good application for further wastewater treatment.

## Abstract 2

In this study, the removal performance for rhodamine B (RB) by persulfate (PS) activated by the CuFe_2_O_4_ catalyst in a heterogeneous catalytic system under LED light irradiation was investigated.

## Graphical abstract

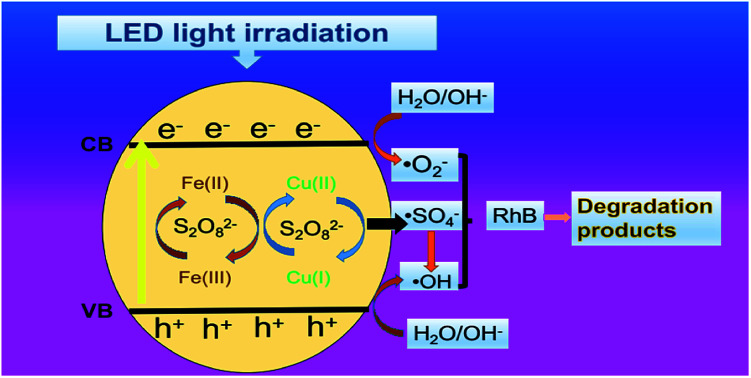

## 1. Introduction

During the past few decades, advanced oxidation processes (AOPs) have attracted significant attention, and many efforts have been made to enhance the degradation efficiency of materials to eliminate bio-refractory organic pollutants.^1^ Notably, the use of sulfate radical (·SO_4_^−^)-based AOPs is an emerging strategy that has obtained widespread application in the mineralization of organic contaminants such as dyes,^2–4^ antibiotics,^5–7^ and phenolic compounds.^8–10^ Compared to hydroxyl radicals, sulfate radicals possess higher standard reduction potential and longer half-life time in solution, which can result in more efficient degradation over a wide pH range (2–11).^11–13^ Sulfate radicals can be simulated by the activation of persulfate using various approaches, heat,^14^ UV light,^15^ and transition metals.^16–18^ However, owing to the restricted pH range (almost around 3.0) and secondary sludge pollution in homogeneous systems, heterogeneous catalysts have been developed as alternatives, exhibiting high activation of persulfate and minimizing the leaching of metal ions.^19^

Among the activation metal catalysts, iron-based catalysts are widely utilized for the activation of persulfate due to the low cost, non-toxicity and natural existence of iron in the environment;^20–22^ metal spinel ferrite is one type of promising heterogeneous catalyst, which presents good activation of persulfate and is easily separated from water due to its spinel structure and magnetic properties. Moreover, it has been reported that the application of metal spinel ferrite with the addition of persulfate in heterogeneous systems under visible light significantly enhances the degradation efficiency of contaminants.^23–25^ Therefore, the development of powerful, economic and environmentally friendly visible light-driven catalysts remains important.

Copper ferrite (CuFe_2_O_4_) has attracted significant attention as a heterogeneous catalyst in the activation of persulfate due to the transition of Cu(i)/Cu(ii) and Fe(ii)/Fe(iii) on the catalyst surface. On the other hand, copper ferrite is recognized as a potential photocatalyst for the activation of persulfate, with a narrow band gap ranging between 1.9 and 2.3 eV, which has shown notable photocatalytic activities in catalyst/PS or catalyst/H_2_O_2_ systems under light irradiation.^26–28^ In the presence of light irradiation, the efficiency is improved *via* the reduction of transition metals, photogenerated holes and electrons on the CuFe_2_O_4_ surface, facilitating the formation of reactive free radicals in the reaction. Consequently, it is reasonable to assume that the degradation efficiency and rate constant in the CuFe_2_O_4_/PS system will be accelerated under visible light irradiation.

To date, the degradation mechanism of the heterogeneous persulfate system under visible light irradiation still needs further elucidation. The combination of photocatalysis and sulfate radical-based AOPs will not only effectively minimize the recombination chance between photogenerated holes and electrons, but also increase the number of reactive oxidant species, leading to a higher degradation efficiency. The work on CuFe_2_O_4_-activated persulfate under light emitting diode (LED) light is scarce, which can be considered a promising alternative to the conventional UV light source due to its high conversion efficiency, narrow band emission and long usage time. Moreover, to the best of our knowledge, few works have focused on the application of CuFe_2_O_4_ for the degradation of organic pollutants and activation of persulfate under LED light irradiation.

In this work, visible LED light was introduced in the CuFe_2_O_4_/PS system to improve its catalytic activity for the removal of RB. RB was chosen as the target compound due to its toxicity and wide application in numerous industries.^29^ Firstly, it is important to demonstrate that the CuFe_2_O_4_/PS/LED system is efficient and environmentally friendly to remove RB in solution. The CuFe_2_O_4_ catalyst was synthesized through the co-precipitation method. The key parameters for the removal of RB were investigated, such as catalyst dosage, persulfate dosage, initial pH, co-existing anions, and initial RB concentration. Secondly, as previous reported, sulfate and hydroxyl radicals play a unique role in heterogeneous sulfate-based systems.^30–32^ However, in photocatalytic reactions, photogenerated holes and electrons also dominate the reaction. Therefore, the possible catalytic mechanisms for the activation of persulfate by the CuFe_2_O_4_ catalyst under LED irradiation were proposed based on various characterization analysis, radical quenching experiments and EPR tests. By using GC/MS technology to identify the intermediates in the reaction, the proposed RB degradation pathway was investigated. This study provides a fundamental understanding and support for the application of CuFe_2_O_4_/PS/LED systems in wastewater treatment.

## 2. Experimental

### 2.1 Synthesis and characterization of CuFe_2_O_4_ catalysts

All chemicals and reagents were of analytic grade and used without further purification. Magnetic CuFe_2_O_4_ was prepared *via* the co-precipitation method. A certain amount of Cu(NO_3_)_2_·6H_2_O and Fe(NO_3_)_3_·9H_2_O (Sinopharm Chemical Reagent Co., Ltd, China) were dissolved in 10 mL deionized water, where the molar ratio of Cu to Fe reached to 1 : 2. The mixed solution was added to the NaOH (4 M) solution under vigorously stirred for 2 h. The dark mixture was transferred to a 50 mL Teflon-lined stainless autoclave and heated at 160 °C for 24 h. Then the autoclave was cooled to room temperature, and the suspension was washed with deionized water and ethanol, respectively. Finally, the solid was dried at 60 °C for 10 h. Simultaneously, CuO and Fe_2_O_3_ catalysts were also synthesized *via* a similar procedure without the addition of iron nitrate or copper nitrate.

Powder X-ray diffraction (XRD) pattern analysis was performed using a Quantachrome/NOVA 2000X-ray diffractometer coupled with graphite monochromatic Cu Kα radiation (*λ* = 1.54 Å) at an accelerating voltage of 40 kV and current of 30 mA in the 2*θ* range of 10–80°. Photoelectron spectroscopy (XPS) analysis was performed using an X-ray photoelectron (Thermo Fisher ESCALAB250Xi) spectrometer with a monochromatized Al–K X-ray source. UV-vis spectra were obtained on a Shimadzu UV-3600 Plus instrument. The morphology and chemical composition of the catalyst were characterized using a SIGMA 500/VP (ZEISS) scanning electron microscope (SEM) equipped with an energy dispersive spectrometer (EDS). High-resolution transmission electron microscopy (HR-TEM) images were obtained on a JEM-2100F (Japan) transmission electron microscope. N_2_ adsorption–desorption analysis and pore size distribution were measured on a TriStar II 3020 instrument. Magnetic curve measurements were performed with a SQUID-VSM instrument at room temperature.

### 2.2 Photocatalytic activity experiments

Batch experiments were carried out in a 50 mL cylindrical reactor at room temperature. An LED lamp (30 W, 460 nm, Xujia Company, China, lamp spectrum showed in Fig. S2†) was used in all experiments. The initial pH was adjusted using 0.1 M H_2_SO_4_ or 0.1 M NaOH. In each stage, a certain amount of catalyst was suspended in 50 mL RB (Sinopharm Chemical Reagent Co., Ltd, China) solution for 30 min and stirred to achieve absorption equilibrium in the dark. Then, a certain amount of persulfate (DaMao Company, China) was added and the LED lamp (the experimental set-up is shown in Fig. S1†) was turned on, which was warmed up for 30 min to reach a steady irradiation efficiency. 1 mL aliquots were regularly withdrawn and filtered through a 0.22 μm membrane, which were quenched with methanol before analysis. The experiments were repeated three times and mean values were used, which had a standard deviation of no more than 3%.

### 2.3 Analysis methods

The concentration of RB was measured by the absorbance in the UV-visible spectrum at the characteristic wavelength of RB (*λ*_max_ = 554 nm) using a UV-vis spectrophotometer (UV3600 II, Shanghai, China). The metal leaching measurements were performed using an ICP-MS (Agilent 7000). The mineralization of RB was performed using a total organic carbon (TOC) analyser (Elementar Vario) after the sample was quenched by methanol. The pH was monitored with a pH meter (Shanghai LeiCi PHS-25) equipped with a pH electrode. Electron spin resonance (ESR, JES FA200, JEOL) was used to measure the intensity of free radicals in methanol and deionized water. The degradation products were detected by GC/MS, using an Agilent 7890B gas chromatograph equipped with an Agilent 7000C mass spectroscopy instrument.

1 Decolorization efficiency (%) = (1−*C*/*C*_0_) × 100%

where *C* represents the concentration of RB at time *t* and *C*_0_ the initial RB concentration.

## 3. Results and discussion

### 3.1 Removal of RB in different systems

**Fig. 1:**
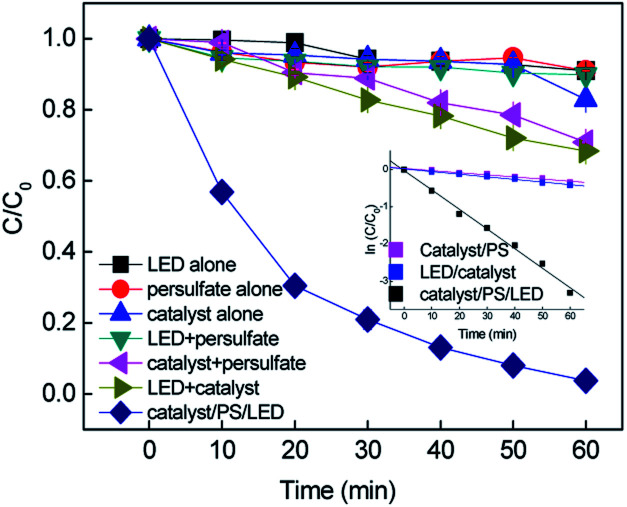
Removal of RB in the different systems and the inset shows the kinetic curves (*C*_0_ = 20 mg L^−1^, [catalyst] = 0.5 g L^−1^, [PS] = 0.2 mM, neutral pH).

Batch-controlled experiments were performed to evaluate the removal efficiency of RB in different systems. As shown in Fig. 1, more than 96% removal of RB was achieved in 60 min by the CuFe_2_O_4_/PS/LED system. The adsorption of RB on the CuFe_2_O_4_ catalyst and use of LED light alone resulted in limited removal efficiency under these experimental conditions, indicating the RB molecule was stable under visible LED light irradiation. As shown in Fig. S6,† the specific area of the synthesized CuFe_2_O_4_ was 12.1 m^2^ g^−1^, with a pore diameter of 6.8 nm, which is responsible for the limited adsorption ability. Negligible colour removal was obtained using persulfate alone due to the limited persulfate decomposition without activation. The combination of persulfate and LED light resulted in 10.11% removal of RB, which indicates persulfate can hardly be activated to produce free radicals under LED light. However, the CuFe_2_O_4_/PS system exhibited 29.17% removal of RB, implying the persulfate can be activated by CuFe_2_O_4_ to obtain free radicals (eqn. (2) and (3)). The oxidation of RB was not completed under the LED/CuFe_2_O_4_ system, which resulted in 34.16% removal efficiency owing to the photogenerated holes and electrons produced in the reaction with sequenced reactive oxidant free radicals (eqn. (4)–(6)).^33–35^ The generated reactive oxidant species reacted with RB, leading to the degradation process.

2 S_2_O_8_^2−^ + Fe(ii) → ·SO_4_^−^ + SO_4_^2−^ + Fe(iii)

3 S_2_O_8_^2−^ + Cu(i) → ·SO_4_^−^ + SO_4_^2−^ + Cu(ii)

4 CuFe_2_O_4_ + LED light → h^+^ + e^−^

5 h^+^+ H_2_O → H^+^ + ·OH

6 e^−^ + O_2_ → ·O_2_^−^

As expected, an obvious improvement in the rate constant was obtained upon the combination of CuFe_2_O_4_ with persulfate under visible LED light irradiation. To evaluate the synergistic effect of the CuFe_2_O_4_ catalyst, CuO and Fe_2_O_3_ catalysts were synthesized to evaluate the activating performance of PS. The CuO and Fe_2_O_3_ catalysts coupled with PS under LED irradiation slightly enhanced the degradation efficiency, yielding an RB removal of 35.4% and 41.1%, respectively. During the process, the persulfate oxidants could react with Fe and Cu repeatedly due to the transition of Fe and Cu on the CuFe_2_O_4_ surface. The kinetics for the removal of RB fitted well with the pseudo-first-order reaction, and the rate constants were determined to be 0.0057, 0.007 and 0.0525 min^−1^ in the CuFe_2_O_4_/PS, CuFe_2_O_4_/LED and CuFe_2_O_4_/PS/LED systems, respectively. The addition of LED light to the catalyst/PS systems enhanced the RB removal. Consequently, the catalyst/PS combined with LED light showed the best removal efficiency for RB under certain conditions.

### 3.2 Effect of parameters on catalytic performance

To obtain the optimized reaction parameters, different operation parameters were carried out. The removal kinetics of RB were demonstrated by the pseudo-first-order reaction.

7 ln *C*/*C*_0_ = −*k* × *t*

where *k* is the pseudo-first-order rate constant, *C* is the concentration of RB at time *t*, and *C*_0_ is the initial RB concentration.

#### 3.2.1 The effect of catalyst dosage

**Fig. 2:**
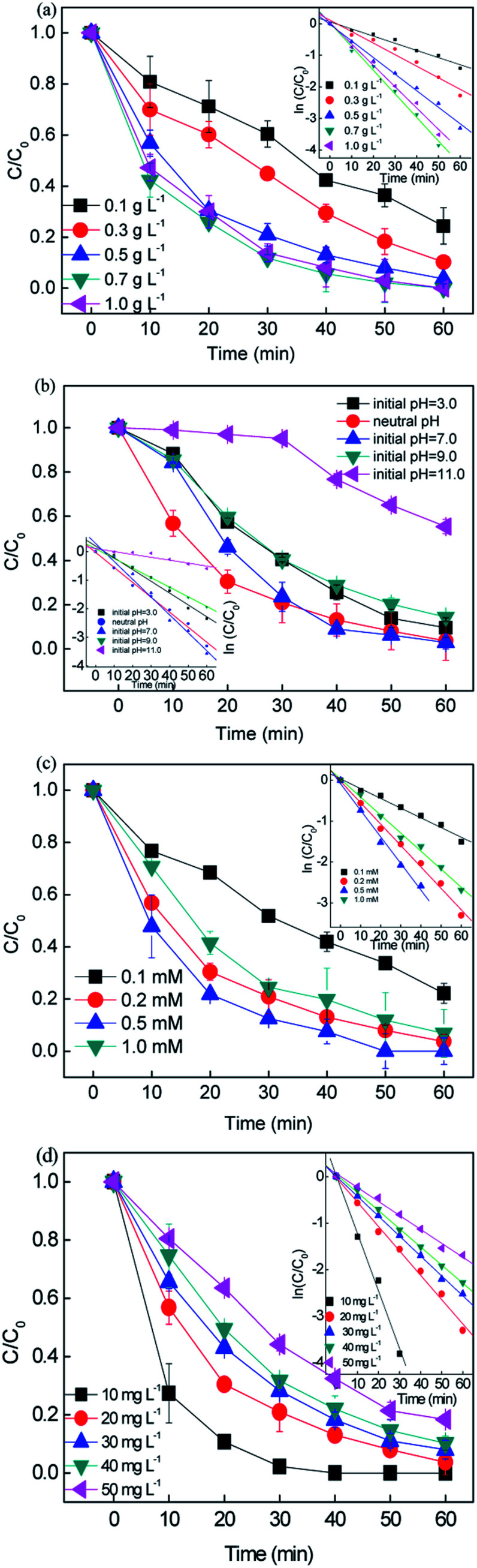
Effect of the reaction conditions on the removal of RB: (a) catalyst dosage, (b) initial pH, (c) persulfate concentration, and (d) initial RB concentration ([catalyst] = 0.5 g L^−1^, [PS] = 0.2 mM, *C*_0_ = 20 mg L^−1^).

The catalyst dosage was investigated in the range of 0.1–1.0 g L^−1^ with the concentration of RB fixed at 20 mg L^−1^ and persulfate fixed at 0.2 mM. The results are depicted in Fig. 2(a). An increase in catalyst dosage resulted in an increase in the removal efficiency to > 99% in 60 min. The rate constant *k* varied from 0.071 to 0.0747 min^−1^, which can be attributed to (i) the increase in the amount of reactive sites provided more surface area and (ii) the increase in available active site generated more active free radicals.^36^ However, a negligible improvement in removal efficiency was obtained when the catalyst dosage was higher than 0.7 g L^−1^ since the rate constant slightly decreased from 0.0747 to 0.0675 min^−1^. With the overdosed catalyst, the number of free radicals increased, and the extra free radicals could not be efficiently utilized and tended to recombination (eqn. (8) and (9)). Moreover, the excess catalyst would hinder light scattering, leading to a reduction in photons.

8 ·SO_4_^−^ + ·SO_4_^−^ → 2SO_4_^2−^

9 ·SO_4_^−^ + Fe(ii) + e^−^ → Fe(iii) + SO_4_^2−^

Consequently, the high catalyst dosage decayed the removal performance.^37^ Finally, 0.5 g L^−1^ was chosen as the optimal dosage for the subsequent experiments.

#### 3.2.2 Effect of initial pH

Since the solution pH is an essential factor in aqueous reactions, the removal of RB under different pH values in the CuFe_2_O_4_/PS/LED systems was investigated. Fig. 2(b) shows the RB decolorization when the initial pH values were set at 3.0, 5.0, 7.0, 9.0 and 11.0. When the initial pH decreased from pH = 7.0 to acidic pH conditions (pH = 3.0), the decolorization varied slightly from 97.17% to 90.43% since *k*_obs_ changed from 0.0626 to 0.0337 min^−1^. The decolorization performance was hindered under basic conditions (pH = 11.0), which can be explained by two possible mechanisms. Persulfate is considered be more stable under highly acidic conditions rather than decomposed to sulfate radicals, thus delaying the decolorization.^38^ On the other hand, persulfate may go through side reactions and decompose to hydroxyl radicals instead of sulfate radicals (eqn. (10)–(12)).^39^ The generated free radicals are assumed to play a unique role in the removal of RB and maintain a high removal rate. In addition, the oxidation capacity of the generated hydroxyl radicals is weaker than that of the sulfate radicals, leading to a decrease in efficiency under basic conditions. Meanwhile, the number of hydroxyl groups (-OH) on the CuFe_2_O_4_ surface would decrease the oxidation potential and catalytic activity at A high pH value. Thus, the results demonstrate that the CuFe_2_O_4_ catalyst can be applied in a wide range of pH values for the activation of persulfate under LED light irradiation compared to the conventional sulfate radical-based systems, which exhibit a better performance under acidic conditions.

10 ·SO_4_^−^ + OH^−^ → SO_4_^2−^ + ·OH

11 ·SO_4_^−^ + ·OH → HSO_4_^−^ + 0.5O_2_

12 S_2_O_8_^2−^ + H_2_O → 2HSO_4_^−^ + 0.5O_2_

#### 3.2.3 Effect of persulfate and RB concentration

Fig. 2(c) shows the effect of persulfate concentration on the removal of RB in the range of 0.1–1 mM. When the persulfate concentration increased from 0.1 mM to 0.2 mM, the removal efficiency increased from 77.8% to 96.75% and the reaction rate constant improved from 0.0238 to 0.0525 min^−1^ during 60 min reaction. The persulfate plays the role of an oxidant in this process, and with an increase in oxidant, the generation of free reactive radicals will be accelerated, leading to a higher RB removal efficiency. However, a further increase in persulfate concentration to 1 mM did not result in a higher removal efficiency. This was caused by the side reactions among the persulfate and free radicals, which would be dominant when excess persulfate is present. The free radicals would prefer to react with the extra PS than RB, leading to the consumption of free radicals and generation of weak reactive species, *i.e.*·S_2_O_8_^−^.^40^ Finally, the concentration of persulfate was chosen to be 0.2 mM for the following experiments owing to the economical consideration.

13 S_2_O_8_^2−^ + ·SO_4_^−^ → ·S_2_O_8_^−^ + SO_4_^2−^

14 S_2_O_8_^2−^ + ·OH → ·S_2_O_8_^−^ + OH^−^

15 ·OH + ·OH → H_2_O_2_

16 S_2_O_8_^2−^ + e^−^ → ·SO_4_^−^ + SO_4_^2−^

17 Fe(iii) + Cu(i) → Fe(ii) + Cu(ii)

The effect of initial RB concentration on the process performance was studied in Fig. 2(d). It can be seen that with an increase in the initial RB concentration from 10 to 50 mg L^−1^, the RB removal decreased from 100% to 81.6% in 30 min, the corresponding removal rate constant dropped from 0.119 to 0.03 min^−1^. This may be because the number of generated free radicals was constant with a fixed CuFe_2_O_4_ catalyst and persulfate concentration. As a result, the increase in RB concentration resulted in a relative reduction in reactive species towards RB molecules. Moreover, the high concentration of by-products at a high RB concentration would compete with the RB molecules, leading to a decrease in the removal efficiency. Consequently, a higher initial RB concentration obviously induced a decrease in the removal efficiency for constant free radicals during the removal process.

#### 3.2.4 Effect of co-existing anions

Since the inorganic anions Cl^−^, NO_3_^−^ and HCO_3_^−^ usually exist in natural water, RB removal by adding the above inorganic anions in different concentrations was explored. It can be seen that the inorganic anions showed different effects on the removal efficiency. Compared to the system without NO_3_^−^, the RB removal efficiency showed slight changes with the addition of 1, 10, and 50 mM NO_3_^−^, while the rate constant decreased from 0.0413 to 0.0261 min^−1^. In the presence of NO_3−_, a reaction between NO_3_^−^ and ·SO_4_^−^, ·OH occurred and formed radicals ·NO_3_ (2.3 eV) and ·NO_2_ (1.03 eV), which had a lower oxidant potential, leading to a decrease in the removal efficiency (eqn. (18)–(20)).^41^

18 ·HO + NO_3_^−^ → OH^−^ + ·NO_3_

19 ·SO_4_^−^ + NO_3_^−^ → ·NO_3_ + SO_4_^2−^

20 ·NO_3_ + H_2_O + e^−^ → ·NO_2_ + 2OH^−^

**Fig. 3:**
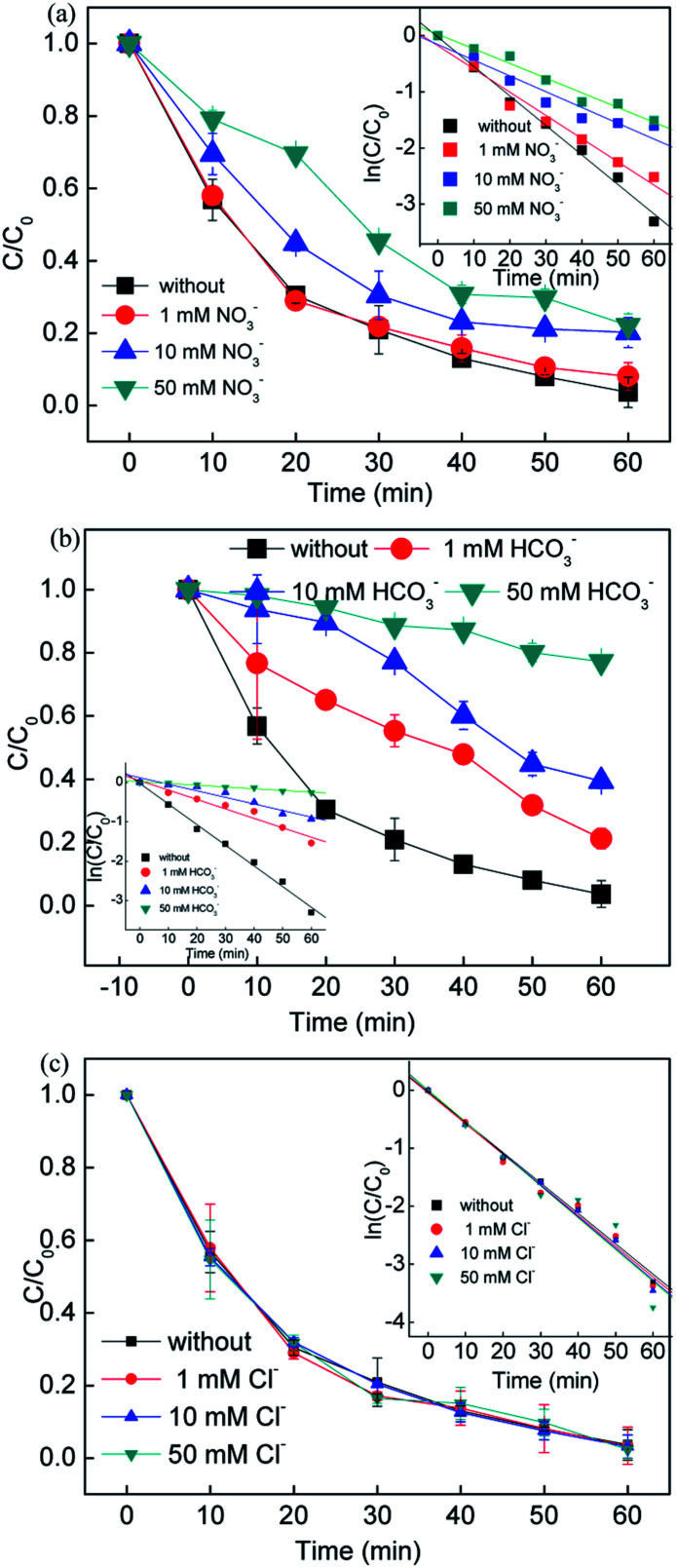
Influence of inorganic anions on the removal of RB. Reaction conditions: [catalyst] = 0.5 g L^−1^, [PS] = 0.2 mM, *C*_0_ = 20 mg L^−1^.

Fig. 3(b) shows that the removal efficiency was > 97%, 78.75%, 60.59% and 22.75% with 0, 1, 10, and 50 mM HCO_3_^−^ added in the solution, respectively, while the corresponding rate constant also dramatically dropped from 0.0525 to 0.0045 min^−1^. It was assumed that the HCO_3_^−^ ions would compete with the RB molecules since the free radicals tended to undergo side reactions with the formation of less active radicals (*i.e.* ·CO_3_^−^ and ·HCO_3_^−^) (eqn. (21) and (22)).^42^ The added HCO_3_^−^ ions played a negative role on the RB removal due to the free radicals scavenged. On the other hand, the free radicals would be consumed very quickly with the addition of HCO_3_^−^ ions, leading to negative inhibition in the systems.

21 ·SO_4_^−^ + HCO_3_^−^ → ·HCO_3_ + SO_4_^2−^

22 ·OH + HCO_3_^−^ → ·CO_3_^−^ + OH^−^ + H^+^

Different dosages of Cl^−^ ions were added to the reaction solution to evaluate the influence of Cl^−^ ions on the RB removal. An increase in RB removal efficiency was obtained when the Cl^−^ ion concentration increased from 1 to 50 mM, and the rate constant increased from 0.0529 to 0.0551 min^−1^. It is known that Cl^−^ ions react with sulfate radicals to form chlorine radicals (*i.e.* ·Cl and ·Cl_2_^−^) (eqn. (23)–(25)). Compared with sulfate and hydroxyl radicals, chlorine radicals are considered less reactive but more selective to attack the electron clusters groups, leading to a slight decrease in removal efficiency at the beginning of the reaction (10 min). On the other hand, the generation of the active chlorine species HClO would also reactive the system, improving the removal efficiency.

23 Cl^−^ + ·OH → ·HOCl^−^

24 ·SO_4_^−^ + Cl^−^ → SO_4_^2−^ + ·Cl

25 ·Cl + Cl^−^ → ·Cl_2_^−^

### 3.3 TOC analysis and recycle tests

To investigate the removal of RB in the systems, the TOC removal for the experiments was performed. The TOC removal was 18.79% and 38.79% in the CuFe_2_O_4_/PS/LED system in 30 min and 60 min, while the RB removal was 96% at the same time owing to its partial degradation in the system instead of complete mineralization (Fig. S3(a)†). This is attributed to the decrease in photosensitivity after the selected irradiation time with the generation of small organic molecules. Moreover, it is assumed that the added persulfate was lower than the calculated theoretical stoichiometry for the complete mineralization of 20 mg L^−1^ RB solution, leading to its incomplete mineralization. The catalytic performance of the heterogeneous CuFe_2_O_4_/PS/LED system was also verified with other organic pollutants, such as levofloxacin (LVX), Orange II, phenolic, ciprofloxacin (CIP), and methyl red (MR). As shown in Fig. S3(b),† the degradation efficiency of the organic pollutants was quite different, approximately 24.1% for LVX, 22.1% for CIP, 90.8% for Orange II, 74.8% for MR, and 68.3% for phenolic, and almost no further degradation occurred after 60 min reaction. Thus, this heterogeneous system is suitable for the selective removal of organic pollutants from wastewater, and should have good application prospects in wastewater treatment.

**Fig. 4:**
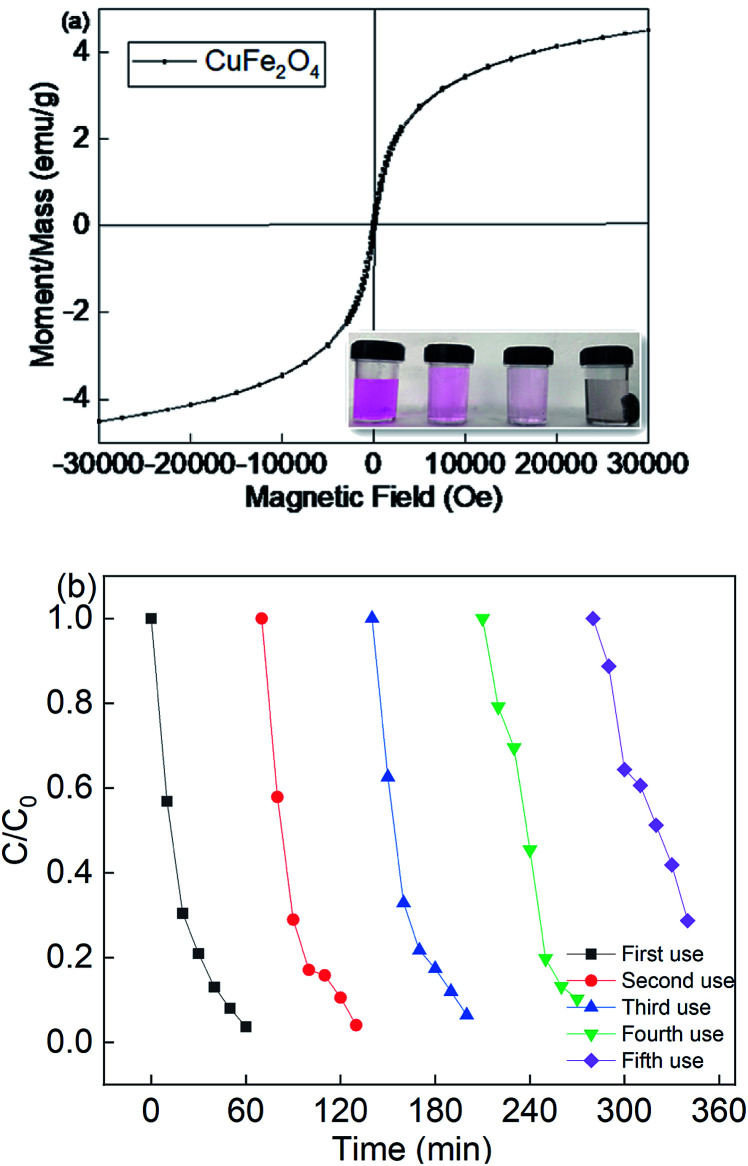
(a) Magnetic hysteresis loops of CuFe_2_O_4_ and (b) reusability of CuFe_2_O_4_ for the removal of RB. Reaction conditions: [catalyst] = 0.5 g L^−1^, [PS] = 0.2 mM, and *C*_0_ = 20 mg L^−1^.

Since the CuFe_2_O_4_ catalyst was recognized to be the heterogeneous catalyst for the persulfate activation, it was important to investigate the reusability of this catalyst. The *M–H* hysteresis loops verified the magnetism of CuFe_2_O_4_. It can be seen that the catalyst was a superparamagnetic material, with the saturation magnetism of 4.55 emμ g^−1^. Fig. 4(a) shows that CuFe_2_O_4_ could be separated from the suspension using a magnet. The results indicate that the saturation magnetism of the magnetic CuFe_2_O_4_ material was enough for it to be separated from water. Therefore, CuFe_2_O_4_ could be used for the reusability test due to its magnetism.

Fig. 4(b) showed that the removal efficiency was almost maintained over multiple cycles, but the rate constant decreased from 0.0525 to 0.0196 min^−1^ due to the recycle use (Fig. S4†). Moreover, the metal leaching under different pH values was also determined, as shown in Fig. S5.† With a decrease in the pH value, the iron and copper leaching was much higher than that under a neutral and basic pH value. The catalyst preparation involved basic conditions, and thus it was less stable under acidic solutions. However, the neither iron nor copper leaching concentration was higher than 0.41 mg L^−1^, indicating that the catalyst showed good catalyst stability. The leaching concentration of Fe and Cu species was 0.15 mg L^−1^ and 0.23 mg L^−1^ at a neutral pH value, respectively. Thus, the homogeneous experiments were also performed at a fixed persulfate dosage. The removal efficiency was about 17.8%, which strongly demonstrates that the reaction system was a heterogeneous catalytic system for the activation of persulfate. The above results indicate that the CuFe_2_O_4_ catalyst possesses good stability and durability for the activation of persulfate under LED light irradiation.

### 3.4 Radical identification and catalysis mechanism

**Fig. 5:**
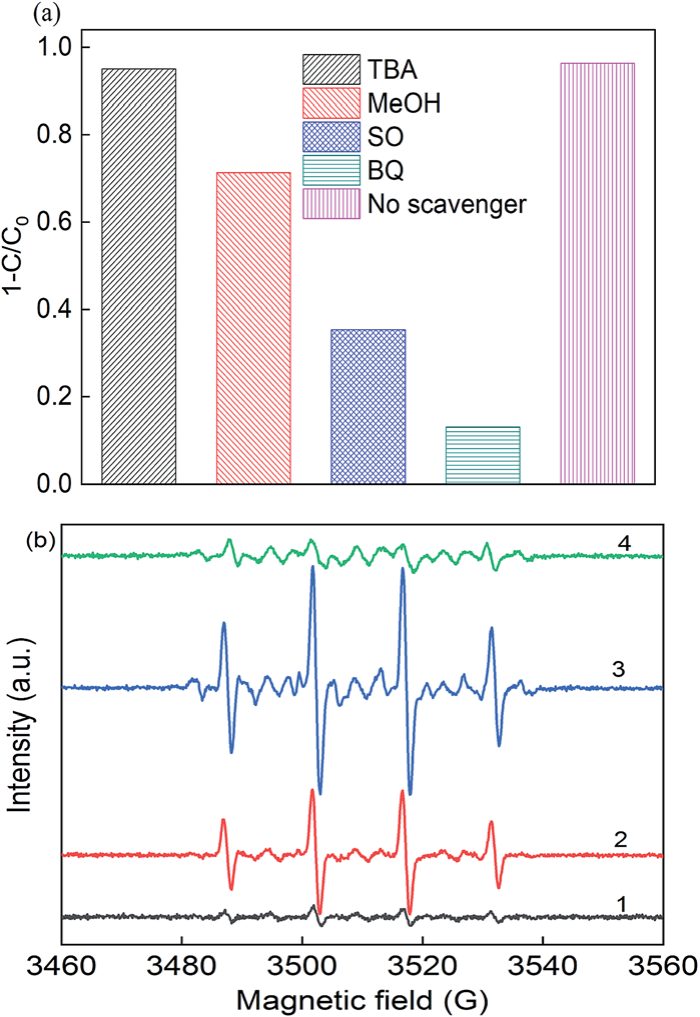
(a) Radical scavengers in the removal of RB and (b) EPR spectra (1) PS alone, (2) in the dark, (3) under LED light in deionized water, and (4) under LED light in methanol solution. Reaction conditions: [catalyst] = 0.5 g L^−1^, [PS] = 0.2 mM, *C*_0_ = 20 mg L^−1^, neutral pH, and [DMPO] = 0.1 M.

In photo-assisted sulfate radical-based systems, reactive sites play a great role in the RB removal efficiency. Thus, to verify the major reactive radicals dominating the reaction, scavenger chemicals were used, including *tert*-butyl alcohol (TBA), methanol (MeOH), sodium oxalate (SO) and *p*-benzoquinone (BQ), as shown in Fig. 5(a). As reported, MeOH is a scavenger for both ·SO_4_^−^ (3.2 × 106 M^−1^ s^−1^) and ·OH radicals (9.7 × 10^8^ M^−1^ s^−1^), where TBA would tend to be more active with ·OH (3.8–7.6 × 10^8^ M^−1^ s^−1^) than ·SO_4_^−^ (4–9 × 10^5^ M^−1^ s^−1^), and thus it is considered as an ·OH scavenger.^3,4,43^ The removal efficiency deceased from 97% to 71.3%, 95.1%, 35.4%, and 13.1% upon the addition of MeOH, TBA, SO and BQ, respectively. In the presence of TBA, the RB removal slightly decreased to 95.1% due to the quenching of hydroxyl radicals, while the removal efficiency was 71.3% with the addition of MeOH. The results certified that the sulfate radicals significantly participated in the photo-assisted catalytic process. The greatest decrease was found upon the addition of SO and BQ, where the removal efficiency dropped to 35.4% and 13.1%, respectively, indicating that the formation of photo-generated holes and electrons played a dominant role in the reaction. Consequently, ·SO_4_^−^, h^+^ and ·O_2_^−^ radicals are of great importance for the removal process.

To identify the free reactive radicals, EPR experiments were conducted using DMPO as a spin trapping agent, as shown in Fig. 5(b). No characteristic peaks were identified in the EPR spectrum in the presence of PS alone, indicating no significant number of radicals occurred without activation. However, when PS and catalyst were both present in the DMPO solution, strong intensity peaks of DMPO-X (X for ·OH, ·SO_4_^−^, and ·O_2_^−^) adducts were observed in the CuFe_2_O_4_/PS system with an intensity ratio of about 1 : 2:2 : 1, which is the typical peak of the DMPO–OH complex.^44^ After 10 min LED light irradiation, the signal intensity of these peaks was almost twice that of the CuFe_2_O_4_/PS system, which had the intensity of 1 : 2:1 : 2:1 : 2:1, indicating the presence of ·O_2_^−^. The EPR signals detected for DMPO-SO_4_ was really weak due to its quick transition to hydroxyl radicals in solution. Thus, the CuFe_2_O_4_ catalyst was able to produce large quantities of free radicals under visible light irradiation.

**Fig. 6:**
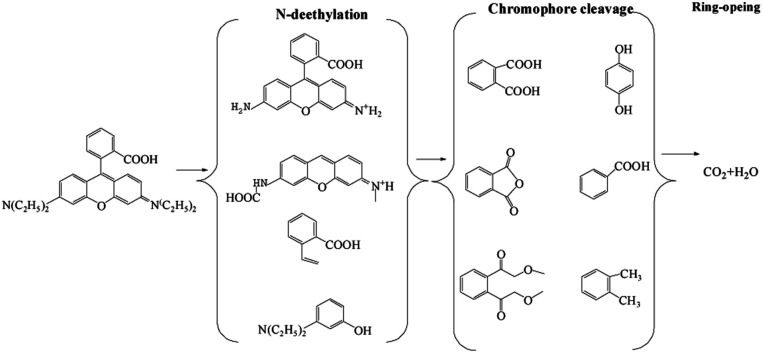
Possible pathway for the photocatalytic degradation of RB in the CuFe_2_O_4_/PS/LED system.

To identify the by-products, the RB molecule was analyzed by GC/MS and the results are shown in Table S1.† The possible pathway for the degradation of RB in the CuFe_2_O_4_/PS/LED process was proposed and shown in Fig. 6, which agreed well with that in previously reported research.^45^ The steps of N-deethylation and chromophore cleavage were considered to occur first in the solution, leading to the decolorization of RB, which is attributed to the generated free radicals attacking the central carbon of RB. Thus, the ring-opening process occurred, resulting in the formation of low-weight molecules in the CuFe_2_O_4_/PS/LED system, such as formic acid, propionic acid, and oxalic acid. Finally, these low weight molecules were further oxidized and turned into CO_2_ and H_2_O.

**Fig. 7:**
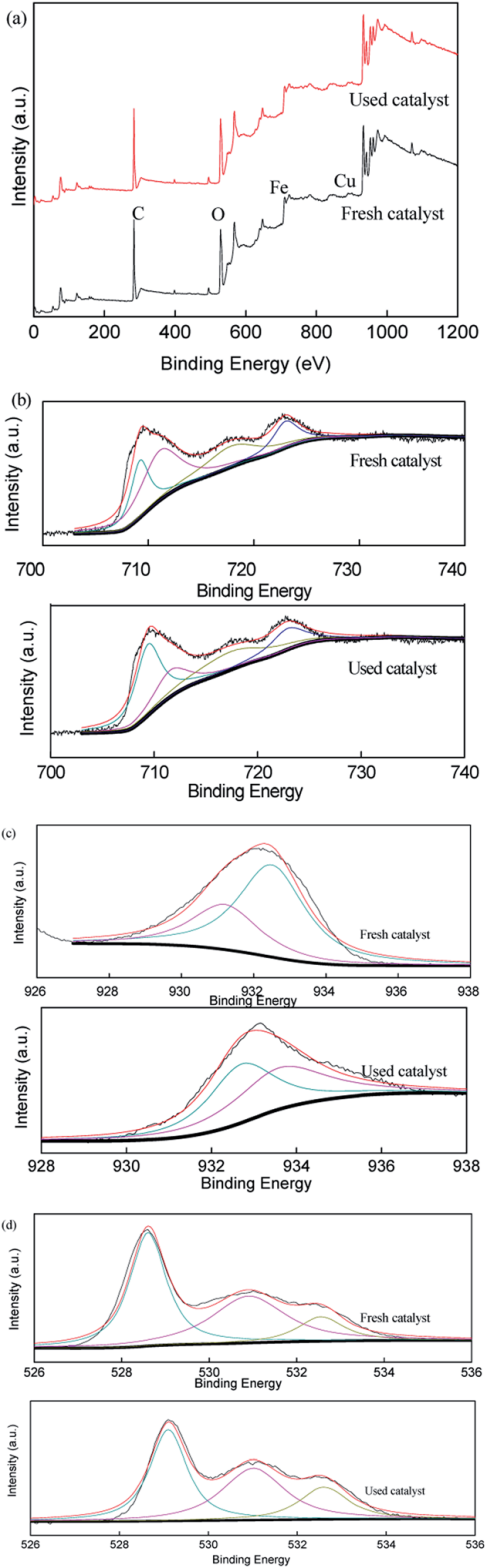
XPS spectra of the fresh and used CuFe_2_O_4_: (a) full range survey, (b) Fe 2p, (c) Cu 2p and (d) O 1s.

To verify the role of transition metal species during the activation of persulfate in the CuFe_2_O_4_/PS/LED system, the XPS spectra of the CuFe_2_O_4_ catalyst before and after the reaction were measured, as displayed in Fig. 7. The representative XPS survey spectra confirmed the presence of Cu, Fe, and O elements. One peak with a binding energy of around 530 eV can be attributed to O 1s, which could be deconvoluted into three internal peaks at 529.7 eV, 531.5 eV and 532.5 eV in the fresh catalyst, representing the lattice oxygen in the metal oxides, hydroxyl groups and absorbed H_2_O on the surface, respectively.^46–48^ The three peaks accounted for 47.8%, 39.5% and 12.7% before the reaction compared to 41.9%, 42.0% and 16.1% after the reaction, respectively, demonstrating the participation of the H_2_O molecule in the reactions and the formation of superoxide radicals, which was more favourable for the transition of Cu(ii)/Cu(i) and Fe(iii)/Fe(ii) under LED light irradiation. The two peaks at 932.5 eV and 933.8 eV in the XPS spectra of Cu are related to Cu 2p_1/2_ and Cu 2p_3/2_, respectively. Meanwhile, the high-resolution spectra peaks of Fe 2p at 711.2 eV and 724.6 eV are indexed to Fe 2p_3/2_ and Fe 2p_1/2_, respectively, indicating that the presence of Fe(iii) occupied most of the iron chemical states before the reaction. However, the Fe(ii) percentage ratio of Fe on the surface of the catalyst increased from 0.29 to 0.38 as Cu(i) increased from 0.49 to 0.67 after reaction with persulfate under LED irradiation. Thus, the results indicate the regeneration of the redox transition metals during the removal process.

**Fig. 8:**
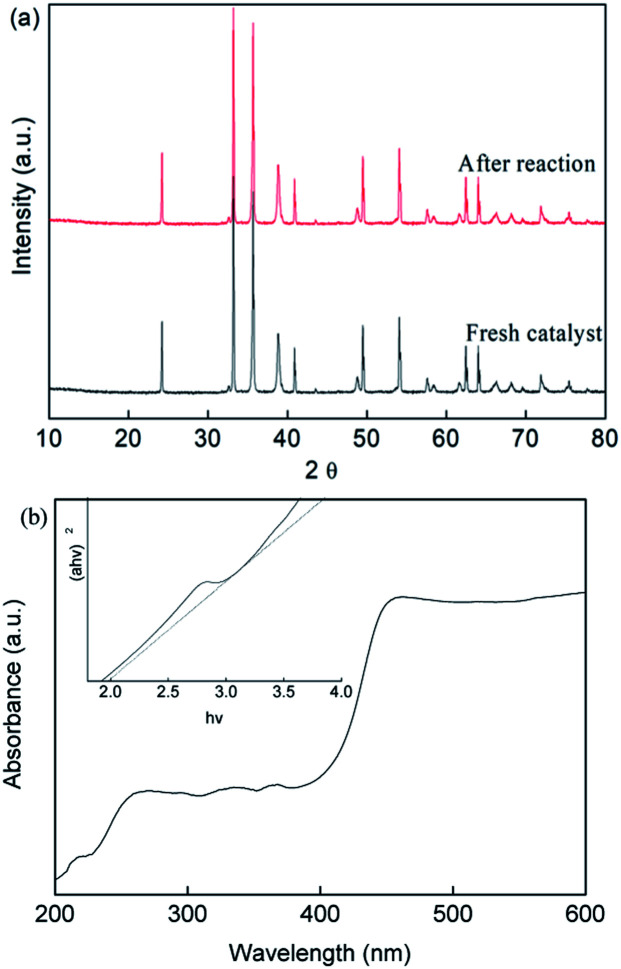
(a) XRD patterns and (b) UV-vis spectra of the CuFe_2_O_4_ catalyst before and after the reaction.

On the other hand, the crystal phase structure of the prepared CuFe_2_O_4_ catalyst before and after the reaction was investigated by XRD measurements, as shown in Fig. 8(a). The main phase of CuFe_2_O_4_ with diffraction peaks is in good agreement with the standard patterns of CuFe_2_O_4_ (JCPDS NO. 34-0425), which had six intense peaks at 2*θ* of 24.2°, 30.3°, 35.8°, 43.73°, 57.95° and 62.58°. These narrow and strong peaks showed the good degree of crystallization of the CuFe_2_O_4_ catalyst, which had an average size of around 53.2 nm according to the Debye–Scherrer formula.

The major phase of the CuFe_2_O_4_ catalyst showed no significant difference between the fresh and used catalyst, indicating the stability of the CuFe_2_O_4_ structure. The UV-vis DRS spectra (Fig. 8(b)) showed that the CuFe_2_O_4_ catalyst possessed a long band absorption in the visible light region. The band gap energy of the semiconductor was calculated to be fixed at 2.01 eV, which is expected to respond to visible LED light irradiation. Upon exposure of the reaction to LED light, the photo-generated electrons and holes were excited from the VB to the CB band, resulting in the generation of holes in the VB band.^49–51^ The *E*_VB_ and *E*_CB_ were calculated using eqn (26) and (27) to be 1.86 V and −0.15 V, respectively.

26 *E*_VB_ = *X* − *E*^Θ^ + 0.5*E*_g_

27 *E*_CB_ = *E*_VB_ − *E*_g_

where *E*_VB_ and *E*_CB_ are the valence band (V) and conduction band (V), respectively, *E*_g_ is the band gap (eV), *E*^Θ^ is the energy of free electrons on the hydrogen scale, which was 4.5 eV, and *X* is the absolute electronegativity of the semiconductor.

**Fig. 9:**
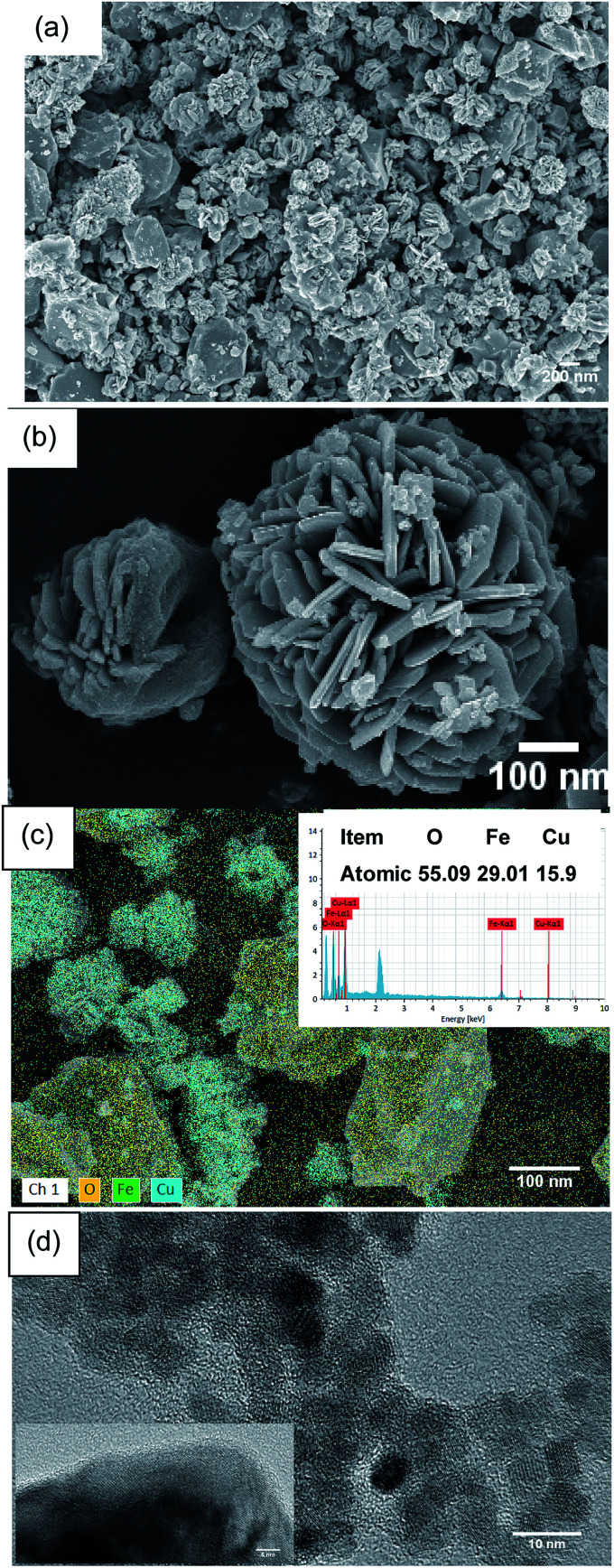
(a) and (b) SEM, (c) EDS mapping and (d) TEM images of the CuFe_2_O_4_ catalyst.

As shown in Fig. 9, the morphology and microstructure of the CuFe_2_O_4_ catalyst were also investigated using SEM and TEM. As can be seen in Fig. 9(a) and (b), the CuFe_2_O_4_ catalyst exhibited a flower-like structure. Fig. 9(c) shows the composition of the fresh CuFe_2_O_4_ catalyst and that the atomic ratio of Cu and Fe was almost close to 1 : 2 in the fresh samples, which is close to the theoretical proportion. The TEM image of CuFe_2_O_4_ showed that the average size of the particles was close to 15 nm, where the particles presented partial agglomeration. In the typical HR-TEM image, the catalyst showed an interplanar distance of 0.241 nm, which confirmed the formation of ferrite nanocrystals (Fig. 9(d)).

**Fig. 10:**
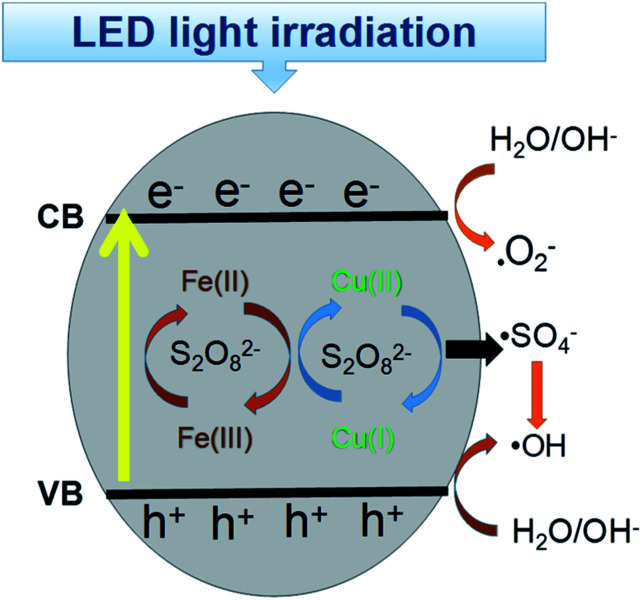
Catalytic mechanism for the degradation of RB in the CuFe_2_O_4_/PS/LED system.

Based on these findings, the probable mechanism for the removal of RB is proposed in Fig. 10. The *E*_CB_ was lower than *E*(S_2_O_8_^2−^/·SO_4_^−^) = 2.06 eV, *E*(O_2_/·O_2_^−^) = −0.046 eV, which facilitated the formation of sulfate radicals by the excited electrons and the formation of ·O_2_^−^.^52^ However, the *E*_VB_ value was much higher than the redox potential of RB, indicating the photo-generated holes will directly oxidize RB. Additionally, on the surface of the CuFe_2_O_4_ catalyst, the initial Cu(ii) and Fe(iii) can participate in the decomposition of persulfate to generate ·SO_4_^−^ and ·OH.^53^ In conclusion, electrons were generated, which jumped from the VB to the CB band with the production of holes under LED light irradiation. With the simultaneous presence of superoxide and free radicals, RB^+^ was produced and participated in the sequence reaction owing to the electron transfer to the catalyst. Thus, in this process, RB was degraded by both ·O_2_^−^, holes and sulfate radicals. Moreover, the continuous redox of Cu(ii) and Fe(iii) accelerated the generation of free radicals and enhanced the process.

## 4. Conclusions

In this study, a ferrite CuFe_2_O_4_ catalyst was prepared and applied in a heterogeneous persulfate system under LED light irradiation. The RB removal followed a pseudo-first-order kinetics pattern. More than 96% of 20 mg L^−1^ RB was removed in 60 min with a fixed catalyst dosage of 0.5 g L^−1^ and persulfate concentration of 0.2 mM at neutral pH. The initial pH had a great influence on the removal of RB. With the increment in the amount of persulfate and catalyst, the removal efficiency improved, but was retarded with their excess addition due to competitive reactions. The presence of HCO_3_^−^ and NO_3_^−^ hardly suppressed the removal efficiency, while the presence of Cl^−^ enhanced the performance. The CuFe_2_O_4_ catalyst exhibited a good catalytic performance and reusability in multiple experiments with low leaching of Cu and Fe ions. The XRD results showed that the structure of the CuFe_2_O_4_ catalyst remained unchanged during the reaction. The intermediates were identified by GC/MS, and a proposed RB degradation pathway was given. Therefore, the CuFe_2_O_4_/PS/LED system has good application for the treatment of recalcitrant wastewater.

## Conflicts of interest

There are no conflicts to declare.

## Supplementary Material

RA-009-C9RA05574F-s001
