# Supplementary material for: A facile heterogeneous system for persulfate activation by CuFe2O4 under LED light irradiation
Source: RSC Adv. 2019 Oct 10;9(55):32328–37. doi: 10.1039/c9ra05574f (PMC9072953; doi:10.1039/c9ra05574f)
Supplement: RA-009-C9RA05574F-s001 [file RA-009-C9RA05574F-s001.pdf]

Electronic Supplementary Material (ESI) for RSC Advances.
This journal is © The Royal Society of Chemistry 2019

Supplementary materials

A facile heterogeneous system for persulfate activation by $CuFe_2O_4$ under LED light irradiation

Xin Zhong, * Xiao-Yu Ye, Di Wu, Kai-Xin Zhang and Wei Huang

Department of Environmental Science and Engineering, Beijng Normal University, Zhuhai, China

*Corresponding author: Xin Zhong. Tel: +86-0756-6126103

E-mail: zhongxin@whu.edu.cn

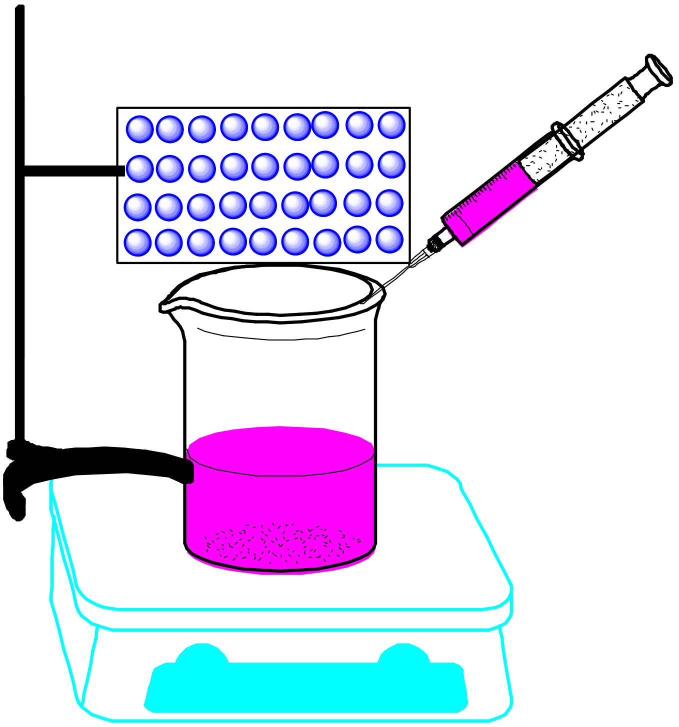

Fig. S1 The experiment set-ups for the experiment

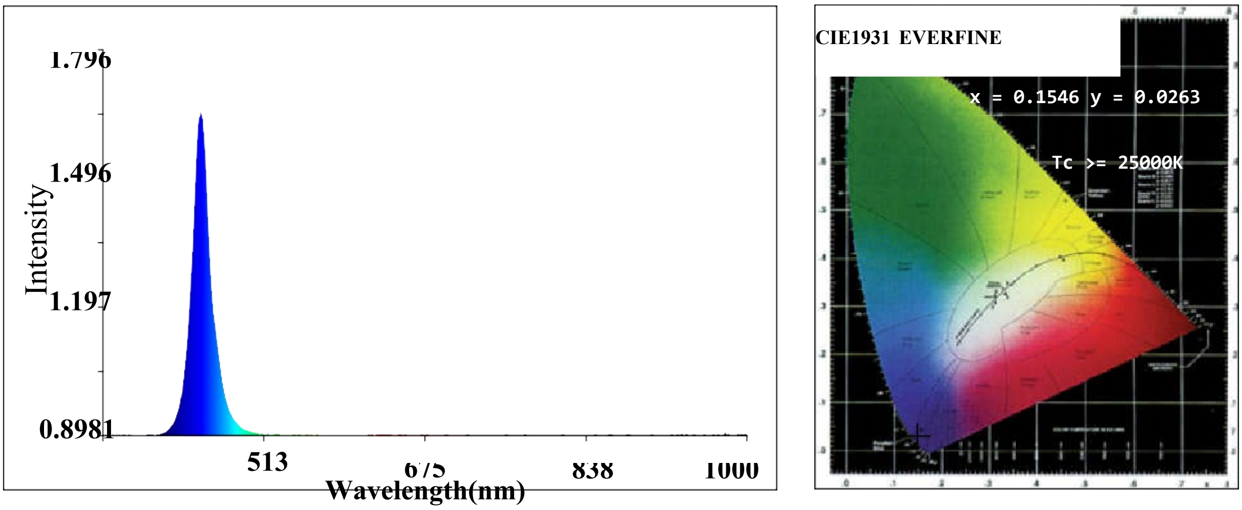

Fig. S2 The photo-luminescence spectra of LED light (Tc = 100000K, λd = 454.1nm, Purity = 98.6%, R = 1.0%, G = 13.1%, B = 86.0%, $\Delta\lambda_d$ = 18.0 nm)

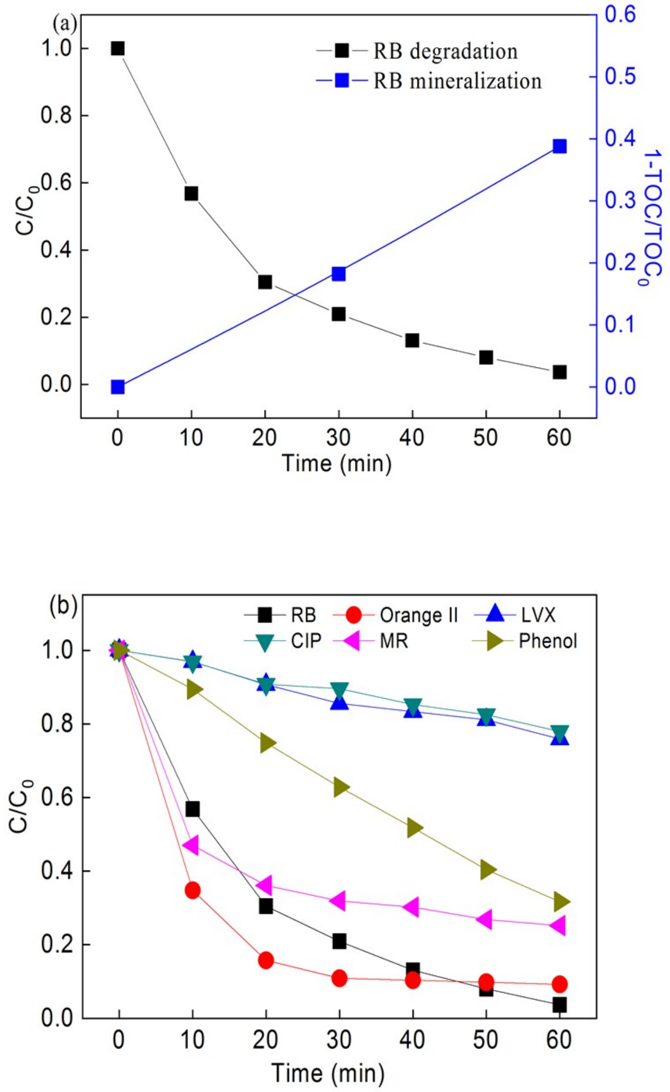

Fig. S3 (a) The degradation and mineralization of RB in $CuFe_2O_4$/PS/LED system, (b) degradation efficiency of various kinds of organic pollutants. Initial conditions: [$CuFe_2O_4$] = 0.5 g L^{-1}, [PS] = 0.2 mM, [organic pollutants]$_0$ = 20 mg L^{-1}, neutral pH.

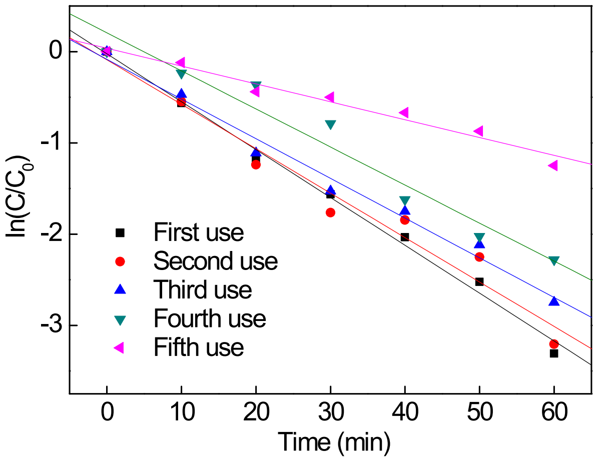

Fig. S4 Reusability of $CuFe_2O_4$ for RB removal kinetic curves. Initial conditions: [$CuFe_2O_4$] = 0.5 g L^{-1}, [PS] = 0.2 mM, C_0=20 mg L^{-1}, neutral pH.

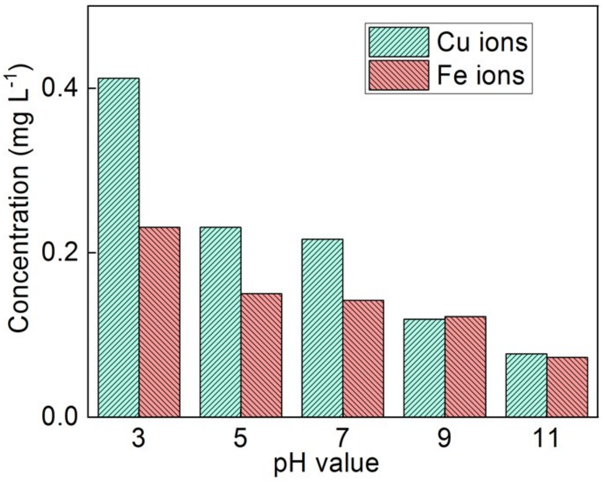

Fig. S5 The iron and copper leaching concentrations under different pH value. Initial conditions: [$CuFe_2O_4$] = 0.5 g L^{-1}, [PS] = 0.2 mM, C_0 = 20 mg L^{-1}.

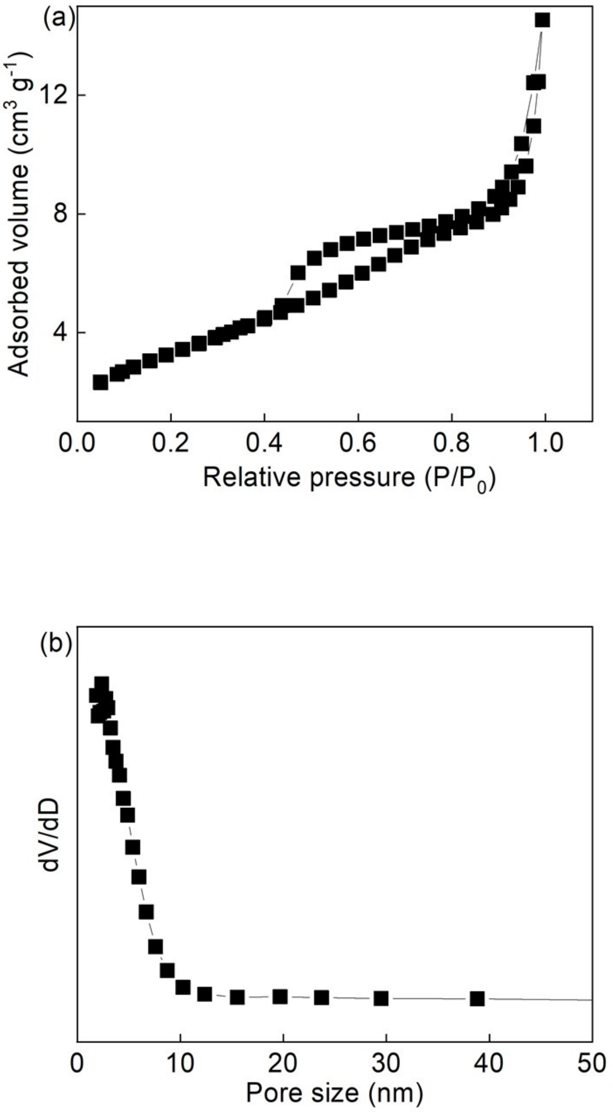

Fig. S6 (a) The N_2 adsorption-desorption isotherms of prepared $CuFe_2O_4$,

(b) pore size distribution.

Table S1 Main reaction intermediates for the degradation of RB in the $CuFe_2O_4$/PS/LED identified by GC/MS analysis.

No.	Indentified intermediates	Structure	m/z
0	Rhodamine B	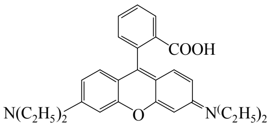	443
1	3-(diethylamino) phenol	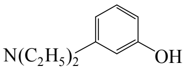	165
2	Phthaic acid	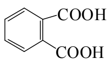	166
3	Isobenzofuran-1,3-dione	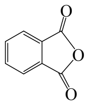	148
4	Methyl hydrogen phthalate	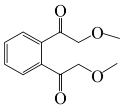	222
5	Hydroquinone	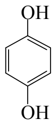	110
6	Benzoic acid	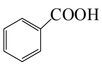	`122
7	o-xylene	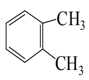	106
